# Preoperative geriatric nutritional risk index as a predictor of postoperative delirium in revision arthroplasty: a 10-year retrospective cohort study

**DOI:** 10.3389/fmed.2025.1626383

**Published:** 2025-07-16

**Authors:** Xuming Chen, Wei Yao, Xiaoyang Liu, Qiyu Xie, Duan Wang, Hong Xu, Zongke Zhou

**Affiliations:** ^1^Department of Orthopedics, Orthopedic Research Institute, West China Hospital, Sichuan University, Chengdu, China; ^2^Department of Orthopedics, Lhasa People's Hospital, Lhasa, China

**Keywords:** geriatric nutritional risk index, postoperative delirium, hip, knee, revision arthroplasty

## Abstract

**Objective:**

This study aims to investigate the association between the preoperative geriatric nutritional risk index (GNRI) and postoperative delirium (POD) in patients undergoing hip or knee revision arthroplasty.

**Methods:**

820 patients who underwent hip or knee revision arthroplasty from January 2014 to September 2024 were included. The exposure variable was preoperative GNRI, and the outcome variable was POD, diagnosed according to the Diagnostic and Statistical Manual of Mental Disorders (DSM-5) criteria using the Confusion Assessment Method (CAM). The study considered covariates such as age, sex, body mass index, albumin, and comorbidities, employing multivariate logistic regression analysis to explore the association between preoperative GNRI and POD.

**Results:**

Among 820 patients, 76 (9.27%) developed POD within 7 days postoperatively. Patients with POD had a significantly lower GNRI (97.53 ± 9.54) compared to those without POD (101.05 ± 8.85, *p* = 0.003). For each 1-unit increase in GNRI, the risk of POD decreased by 4% (OR = 0.96, 95% CI: 0.94–0.99, *p* = 0.011). Quartile analysis showed that patients in the highest GNRI quartile had a significantly lower POD incidence compared to those in the lowest quartile (OR = 0.43, 95% CI: 0.20–0.92, p for trend = 0.037). A protective threshold of GNRI was identified at 101.96.

**Conclusion:**

A significant association was observed between preoperative GNRI and POD in patients undergoing hip or knee revision arthroplasty. However, due to the retrospective single-center design and potential unmeasured confounding, further multicenter prospective studies are warranted to validate these findings and explore underlying mechanisms.

## Introduction

Revision hip and knee arthroplasty represents a substantial and growing clinical and economic challenge. According to recent data from Medicare, between 2000 and 2021, approximately 492,360 revision total knee arthroplasties and 424,163 revision hip arthroplasties were performed in the United States (1). These revision procedures are associated with higher complication rates and morbidity compared with primary joint replacements, including periprosthetic joint infection, prosthetic loosening, dislocation, and postoperative delirium (POD) (2–5). Notably, POD is an acute neuropsychiatric complication that frequently occurs following revision arthroplasty due to prolonged surgical duration, increased complexity, and greater patient frailty (6, 7). It most commonly develops within the first 1–3 days after surgery, although onset can occur at any point during the first postoperative week (8, 9). POD contributes to extended hospital stays, elevated healthcare costs, and heightened postoperative morbidity and mortality (10, 11). Given the heightened clinical risk and economic implications specifically associated with revision procedures, identifying modifiable preoperative risk factors for POD, such as nutritional status, is particularly crucial.

The preoperative geriatric nutritional risk index (GNRI) is an effective tool for assessing the nutritional status of elderly patients. It is more sensitive than the body mass index (BMI) and serum albumin levels in reflecting the nutritional status of older adults (12, 13). GNRI takes into account multiple indicators, including weight, height, and serum albumin, providing an accurate representation of a patient’s overall nutritional condition (14). It is also closely associated with the occurrence of various postoperative complications (15). Recent research consistently indicates that elderly patients with lower GNRI values are more susceptible to developing POD (10, 11, 16), further highlighting the potential value of GNRI in predicting the risk of POD. However, studies examining the association between GNRI and POD in patients undergoing hip or knee revision arthroplasty are still scarce.

We specifically focused on revision arthroplasty because these procedures generally involve greater surgical complexity, longer operative times, and increased blood loss compared to primary surgeries, all of which significantly heighten the risk of POD (17–19). Therefore, accurate identification of modifiable risk factors, such as nutritional status, is particularly critical in this vulnerable patient group.

This study aims to perform a retrospective cohort analysis to investigate the association between preoperative GNRI and POD, particularly in elderly patients undergoing hip or knee revision arthroplasty. By analyzing the clinical data of this population, the research aspires to provide more targeted evidence to the existing literature, further elucidating the role of GNRI in predicting POD, and offering new insights and references for preoperative assessment and management in clinical practice.

## Methods

### Study design and data collection

This study is a retrospective cohort study, with data sourced from electronic medical records at our hospital between January 2014 and September 2024. Data collection was independently conducted by two researchers (XMC and WY), who meticulously cross-checked discrepancies to ensure data accuracy. The study strictly adhered to the ethical principles outlined in the 1964 Declaration of Helsinki and received approval from the Institutional Review Board of West China Hospital, Sichuan University (approval No. 2024–2056). The committee waived the requirement for written informed consent due to the anonymity of patient data, and the study posed no adverse effects on patient health. This study has been registered in the Chinese Clinical Trials Registry (registration No. ChiCTR2500095262).

### Patient selection criteria

**Figure 1:**
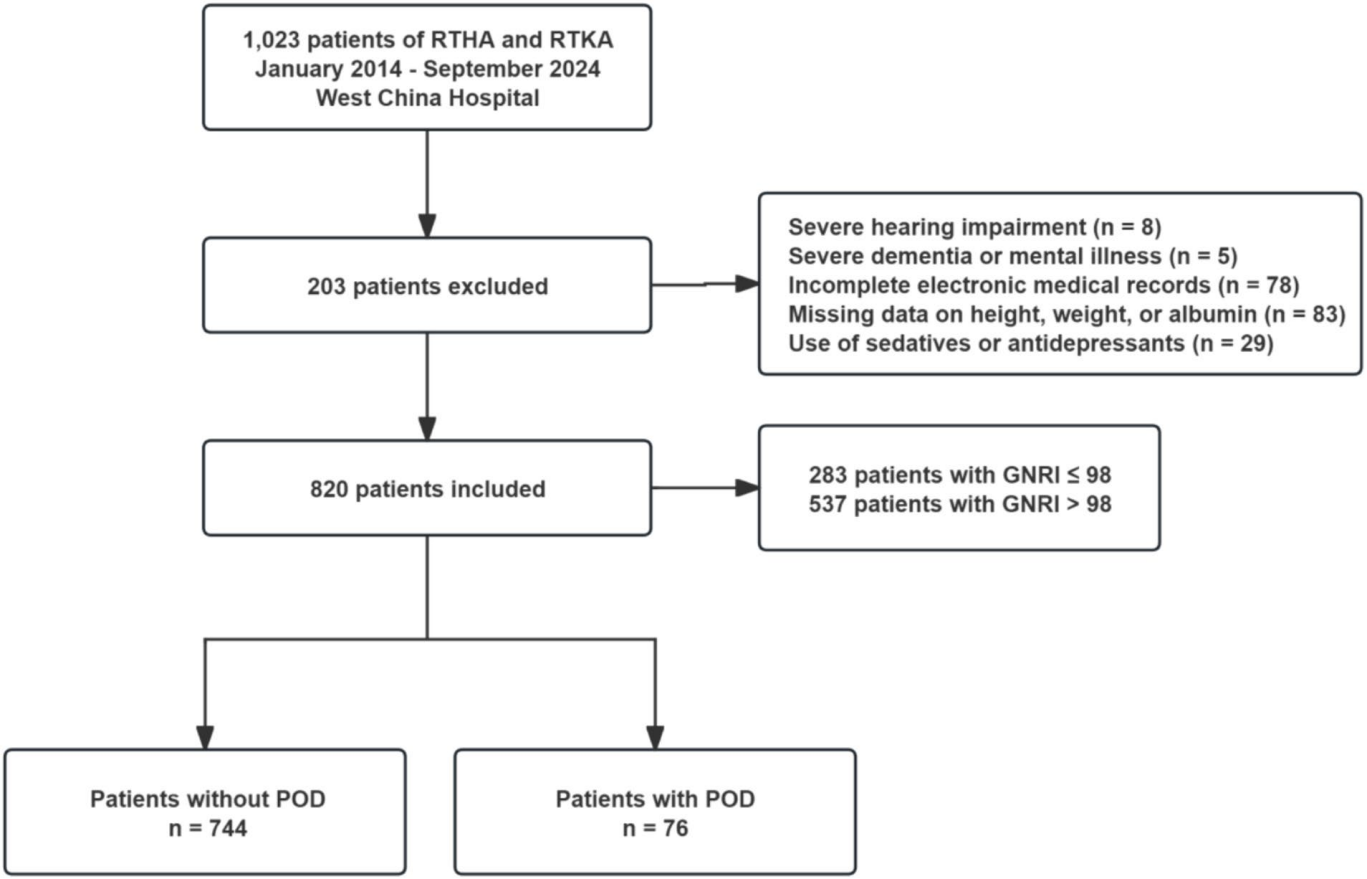
Flow chart of enrollment. RTHA, revisional total hip arthroplasty; RTKA, revisional total knee arthroplasty; GNRI, geriatric nutritional risk index; POD, postoperative delirium.

The study included patients who underwent revision arthroplasty due to PJI, periprosthetic fractures, prosthetic loosening, or dislocation after primary hip or knee arthroplasty. The exclusion criteria included: (1) severe hearing impairment; (2) inability to communicate due to severe dementia or mental illness; (3) incomplete electronic medical records that cannot be accessed; (4) missing baseline data on height, weight, or serum albumin; and (5) use of sedatives or antidepressants during hospitalization. The specific screening process is detailed in Figure 1.

### Exposure variables

The preoperative GNRI is adapted from the nutritional risk index (NRI) designed by Buzby et al. (20). GNRI is a simple and effective nutritional screening tool commonly used to assess nutrition-related postoperative complications. Its calculation formula is (14):


$$\begin{array}{l} \text{GNRI}=1.489\times \text{Serum Albumin}\;(g/L)+41.7\times \\ \;\text{Actual Weight}/\text{Ideal Weight}\;(\mathrm{kg}) \end{array}$$

Serum albumin levels were obtained from routine laboratory testing conducted within 48 h before surgery, as part of the hospital’s standard preoperative assessment protocol.

Ideal weight is calculated using the Lorentz formula (14):


Ideal weight for males=0.75×Height(cm)–62.5

Ideal weight for females=0.60×Height(cm)–40

When the actual weight exceeds the ideal weight, the actual weight/ideal weight ratio is set to 1 (21). Based on preoperative GNRI values, participants were classified into two groups: low GNRI (≤98) and high GNRI (>98), following previously established thresholds in the literature (21–23). Similar to previous studies (24), we further subdivided them into three groups: high GNRI (>98), moderate GNRI (92–98), and low GNRI (<92). To simplify the analysis, severe risk (GNRI < 82) and moderate risk (GNRI 82–92) were combined into one category, as the risk of complications was similar for these two groups (24). Additionally, patients’ GNRI were grouped according to quartiles: Q1 (62.69–95.15), Q2 (95.16–102.00), Q3 (102.01–107.22), and Q4 (107.23–122.70).

### Outcome indicators

Although this was a retrospective study, outcome data-including daily delirium assessments based on the Confusion Assessment Method (CAM) (25), were extracted from structured clinical records maintained during routine inpatient care from the day of surgery through postoperative day 7. These assessments were performed by trained clinical staff as part of routine practice. Two independent research assessors retrospectively reviewed these records and confirmed the diagnosis of POD according to the criteria outlined in the fifth edition of the Diagnostic and Statistical Manual of Mental Disorders (DSM-5) (26), using the CAM algorithm. CAM includes the following criteria: (1) acute onset with a fluctuating course, (2) inattention, (3) disorganized thinking, and (4) altered level of consciousness. A diagnosis of POD requires the presence of both criteria 1 and 2, plus either criterion 3 or 4. Importantly, the assessors were blinded to patients’ preoperative GNRI values throughout the evaluation process to minimize diagnostic bias. Discrepancies between assessors were resolved through discussion and consensus.

### Covariates

Relevant covariates were collected from patients’ medical records based on previously identified risk factors and categorized into four categories: demographic variables, comorbidities, surgery-related variables, and nutritional assessment indicators. Demographic variables included age, sex, BMI, education level, smoking, and alcohol consumption. Comorbidities were assessed using the Agency for Healthcare Research and Quality (AHRQ) Elixhauser Comorbidity Index (ECI) (27), which measures the impact of patient comorbidities on health outcomes and healthcare resource utilization; specific comorbidities are listed in Supplementary Table 1. Surgery-related variables included surgical duration, waiting time from admission to surgery, length of hospital stay, surgical site, and American Society of Anesthesiologists (ASA) score. Nutritional assessment indicators primarily included serum albumin levels and preoperative GNRI.

### Statistical analysis methods

Patient baseline characteristics were described using mean ± standard deviation or numbers (percentages). Missing data were addressed using multiple imputations, controlled within a 5% threshold, to ensure data completeness and accuracy of analyses. For categorical data, chi-square tests were used for comparisons, while continuous data were analyzed using the Kruskal-Wallis test. We analyzed the influence of preoperative GNRI as both a continuous and categorical variable on POD while controlling for potential confounding factors (Supplementary Table 1). All variables underwent model trimming using Akaike information criteria (AIC) (28, 29) to generate a simplified model ensuring that relevant independent variables significantly improved model performance. Specifically, age, sex, surgical duration, length of hospital stay, and surgical site were mandated for inclusion in the model. Further subgroup analyses were conducted to explore potential interactions between covariates. Patients were stratified based on covariates, and independent logistic regression analyses were performed for each subgroup, assessing the presence of interactions by comparing differences in odds ratios (OR) across subgroups. Finally, we used the Receiver Operating Characteristic (ROC) curve and its area under the curve (AUC) to evaluate the diagnostic efficacy of preoperative GNRI in predicting POD. The statistical significance level was set at a two-sided *p*-value < 0.05. All statistical analyses were performed using SPSS Statistics 25.0 for Windows (IBM Corp., Armonk, NY) and R software 4.3.1 for Windows (R Foundation for Statistical Computing, Boston, MA, United States).

## Results

### Baseline characteristics analysis

**Figure 2:**
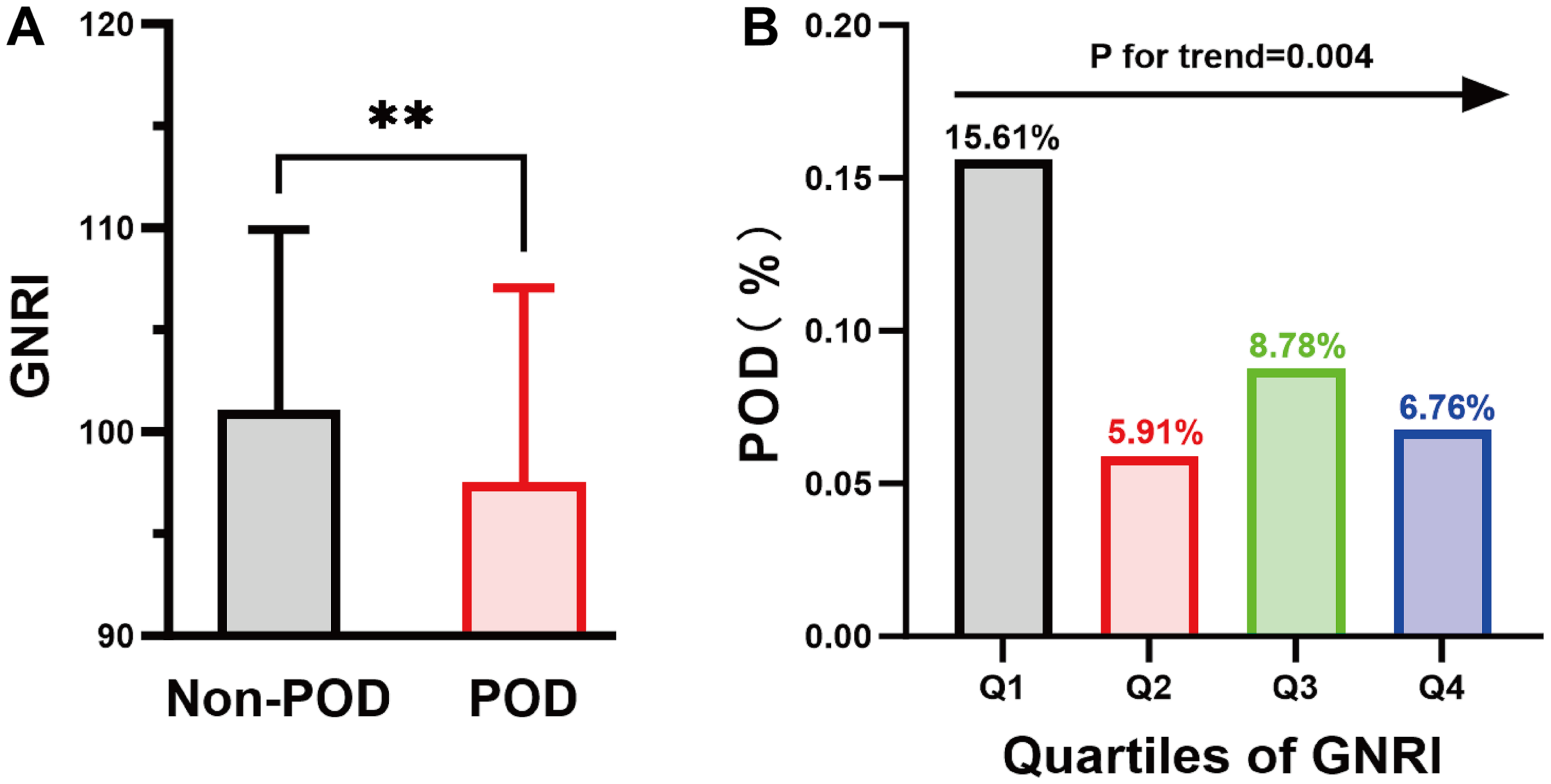
The column chart illustrates the relationship between POD and GNRI values. **(A)** The average GNRI value was 101.05 in the Non-POD group and 97.53 in the POD group. **p < 0.01; **(B)** Highlights the variability in POD incidence across GNRI quartile ranges: 15.61% in the Q1 group, 5.91% in the Q2 group, 8.78% in the Q3 group, and 6.78% in the Q4 group. GNRI, geriatric nutritional risk index; POD, postoperative delirium.

Finally, 820 subjects were included in the analyses. Among them, 283 patients (34.51%) exhibited malnutrition (GNRI ≤ 98), while 76 patients (9.27%) experienced POD (Figure 1). Compared to the non-POD group, the POD group is older, has a lower educational level, and has more comorbidities (Table 1; Supplementary Table 1). In terms of nutritional assessment, the GNRI value for the POD group (97.53 ± 9.54) was significantly lower than that of the Non-POD group (GNRI: 101.05 ± 8.85) (*p* = 0.003) (Table 1). Figure 2A illustrates the difference in GNRI between the two groups, with a particularly pronounced difference observed between the POD and Non-POD cohorts. Baseline characteristics were further stratified by GNRI quartiles to explore their distribution across nutritional status levels (Supplementary Table 2). The visual analysis in Figure 2B indicated that the incidence of POD significantly decreased with an increasing GNRI value (*p* for trend = 0.004).

**Table 1:** Baseline characteristics of the 820 patients.

Characteristics	Total	Non-POD	POD	*p*-value
	*n* = 820	*n* = 744	*n* = 76	
Demographic				
Age, year (Mean ± SD)	61.45 ± 14.32	60.77 ± 14.26	68.11 ± 13.22	<0.001*
Female gender (*n*, %)	442 (53.90)	394 (52.96)	48 (63.16)	0.089
BMI, kg/m^2^ (Mean ± SD)	23.64 ± 3.62	23.68 ± 3.61	23.25 ± 3.66	0.316
Education (*n*, %)				
Illiteracy	50 (6.10)	42 (5.65)	8 (10.53)	<0.001*
Below the junior high school	200 (24.39)	175 (23.52)	25 (32.89)	
Secondary education	518 (63.17)	486 (65.32)	32 (42.11)	
College degree and higher	52 (6.34)	41 (5.51)	11 (14.47)	
Smoking (*n*, %)	189 (23.05)	180 (24.19)	9 (11.84)	0.015*
Alcohol abuse (*n*, %)	170 (20.73)	162 (21.77)	8 (10.53)	0.021*
Comorbidities				
AHRQ ECI (Mean ± SD)	2.03 ± 6.44	1.68 ± 5.95	5.48 ± 9.46	<0.001*
Operation				
Surgery time, hour (Mean ± SD)	2.68 ± 0.67	2.70 ± 0.62	2.50 ± 1.03	0.117
Admission to surgery, day (Mean ± SD)	5.35 ± 4.54	5.46 ± 4.65	4.20 ± 3.13	0.021*
Length of stay, day (Mean ± SD)	13.99 ± 10.35	14.22 ± 10.53	11.76 ± 8.21	0.049*
Surgical site (*n*, %)				
RTHA	658 (80.24)	596 (80.11)	62 (81.58)	0.759
RTKA	162 (19.76)	148 (19.89)	14 (18.42)	
ASA Classification (*n*, %)				
I–II	380 (46.34)	348 (46.77)	32 (42.11)	0.437
III–IV	440 (53.66)	396 (53.23)	44 (57.89)	
Nutritional Assessment Indicators				
Albumin, g/L (Mean ± SD)	40.59 ± 5.59	40.80 ± 5.51	38.61 ± 5.95	0.003*
GNRI (Mean ± SD)	100.72 ± 8.97	101.05 ± 8.85	97.53 ± 9.54	0.003*

RTHA, revisional total hip arthroplasty; RTKA, revisional total knee arthroplasty; POD, postoperative delirium; SD, standard deviation; BMI, body mass index; GNRI, geriatric nutritional risk index; AHRQ, Agency for Healthcare Research and Quality; ECI, Elixhauser comorbidity index; ASA, the American Society of Anesthesiologists physical status classification system. *p < 0.05.

### Multivariate logistic regression analysis

As shown in Table 2, several factors were significantly associated with the incidence of POD. Among them, higher GNRI and longer waiting time from admission to surgery were protective factors (OR < 1), while older age, valvular heart disease, cerebrovascular disease, solid tumors, depression, and venous thromboembolism (VTE) were identified as risk factors (OR > 1). These findings highlight both clinical and nutritional contributors to POD risk. Further multivariate logistic regression analyses revealed a significant association between GNRI and POD, with this association remaining stable after adjusting for various confounding factors. When GNRI was treated as a continuous variable, the results indicated that for every 1-unit increase in GNRI, the risk of POD decreased by 4% (Odds Ratio (OR) = 0.96, 95% Confidence Interval (CI): 0.94–0.99, *p* = 0.011) (Table 3). Additional analyses showed that compared to the low-GNRI group, the odds ratios (OR) for the moderate-GNRI and high-GNRI groups were 0.33 (95% CI: 0.14–0.77) and 0.37 (95% CI: 0.20–0.70), respectively, with p for trend = 0.008. When compared to the first quartile (Q1), the ORs for the second, third, and fourth quartiles were 0.33 (95% CI: 0.15–0.73), 0.66 (95% CI: 0.33–1.31), and 0.43 (95% CI: 0.20–0.92), respectively, with p for trend = 0.037. Therefore, the correlation between preoperative GNRI and POD demonstrated a significantly decreasing trend (trend *p* = 0.008 and *p* = 0.037), suggesting that preoperative malnutrition is an independent risk factor for POD (Table 3).

**Table 2:** Multivariable logistic regression reduced model for the entire cohort.

Variable	OR (95%CI)	*p*-value
GNRI	0.96 (0.94 ~ 0.99)	0.011*
Age (year)	1.03 (1.01 ~ 1.05)	0.012*
Male sex	0.80 (0.46 ~ 1.38)	0.418
BMI (kg/m^2^)	1.01 (0.94 ~ 1.09)	0.753
COPD	2.48 (0.93 ~ 6.63)	0.070
Valvular heart disease	3.62 (1.07 ~ 12.25)	0.038*
Cerebrovascular	6.03 (2.15 ~ 16.88)	<0.001*
Solid-tumor	2.59 (1.04 ~ 6.46)	0.042*
Depression	32.60 (5.56 ~ 191.35)	<0.001*
VTE	2.76 (1.26 ~ 6.03)	0.011*
Surgery time (hour)	0.92 (0.63 ~ 1.33)	0.656
Admission to surgery (day)	0.90 (0.81 ~ 0.99)	0.046*
Length of stay (day)	1.00 (0.96 ~ 1.03)	0.889
Surgical site (hip/knee)	0.85 (0.42 ~ 1.71)	0.644

OR, odds ratio; CI, confidence interval; GNRI, geriatric nutritional risk index; BMI, body mass index; COPD, chronic obstructive pulmonary disease; VTE, venous thromboembolism. *p < 0.05.

**Table 3:** Unadjusted and adjusted associations between GNRI and POD.

GNRI		POD, *n* (%)	Model 1		Model 2		Model 3	
			OR (95%CI)	*p*-value	OR (95%CI)	*p*-value	OR (95%CI)	*p*-value
Continuous	Per 1	NA	0.96 (0.94 ~ 0.98)	0.001*	0.96 (0.93 ~ 0.99)	0.004*	0.96 (0.94 ~ 0.99)	0.011*
Dichotomy	Low GNRI (≤98)	37 (13.07)	1 (Reference)	0.007*	1 (Reference)	0.038*	1 (Reference)	0.072
	High GNRI (>98)	39 (7.26)	0.52 (0.32 ~ 0.84)		0.57 (0.33 ~ 0.97)		0.61 (0.35 ~ 1.05)	
Categories	Low GNRI (<92)	25 (18.66)	1 (Reference)	<0.001*^#^	1 (Reference)	0.003*^#^	1 (Reference)	0.008*^#^
	Moderate GNRI (≥92, ≤98)	12 (8.05)	0.38 (0.18 ~ 0.79)		0.32 (0.14 ~ 0.73)		0.33 (0.14 ~ 0.77)	
	High GNRI (>98)	39 (7.26)	0.34 (0.20 ~ 0.59)		0.34 (0.18 ~ 0.64)		0.37 (0.20 ~ 0.70)	
Quartile	Q1 (62.69–95.15)	32 (15.61)	1 (Reference)	0.004*^#^	1 (Reference)	0.015*^#^	1 (Reference)	0.037*^#^
	Q2 (95.16–102.00)	12 (5.91)	0.34 (0.17 ~ 0.68)		0.33 (0.15 ~ 0.71)		0.33 (0.15 ~ 0.73)	
	Q3 (102.01–107.22)	18 (8.78)	0.52 (0.28 ~ 0.96)		0.60 (0.31 ~ 1.19)		0.66 (0.33 ~ 1.31)	
	Q4 (107.23–122.70)	14 (6.76)	0.39 (0.20 ~ 0.76)		0.38 (0.18 ~ 0.81)		0.43 (0.20 ~ 0.92)	

Model 1: Crude.

Model 2: Adjust: BMI, COPD, valvular heart disease, cerebrovascular, solid-tumor, depression, VTE, admission to surgery.

Model 3: Adjust: BMI, COPD, valvular heart disease, cerebrovascular, solid-tumor, depression, VTE, admission to surgery, age, sex, surgery time, length of stay, surgical site.

GNRI, geriatric nutritional risk index; POD, postoperative delirium; OR, odds ratio; CI, confidence interval; BMI, body mass index; COPD, chronic obstructive pulmonary disease; VTE, venous thromboembolism. *p < 0.05; # *p* for trend.

### Restricted cubic spline curve analysis

**Figure 3:**
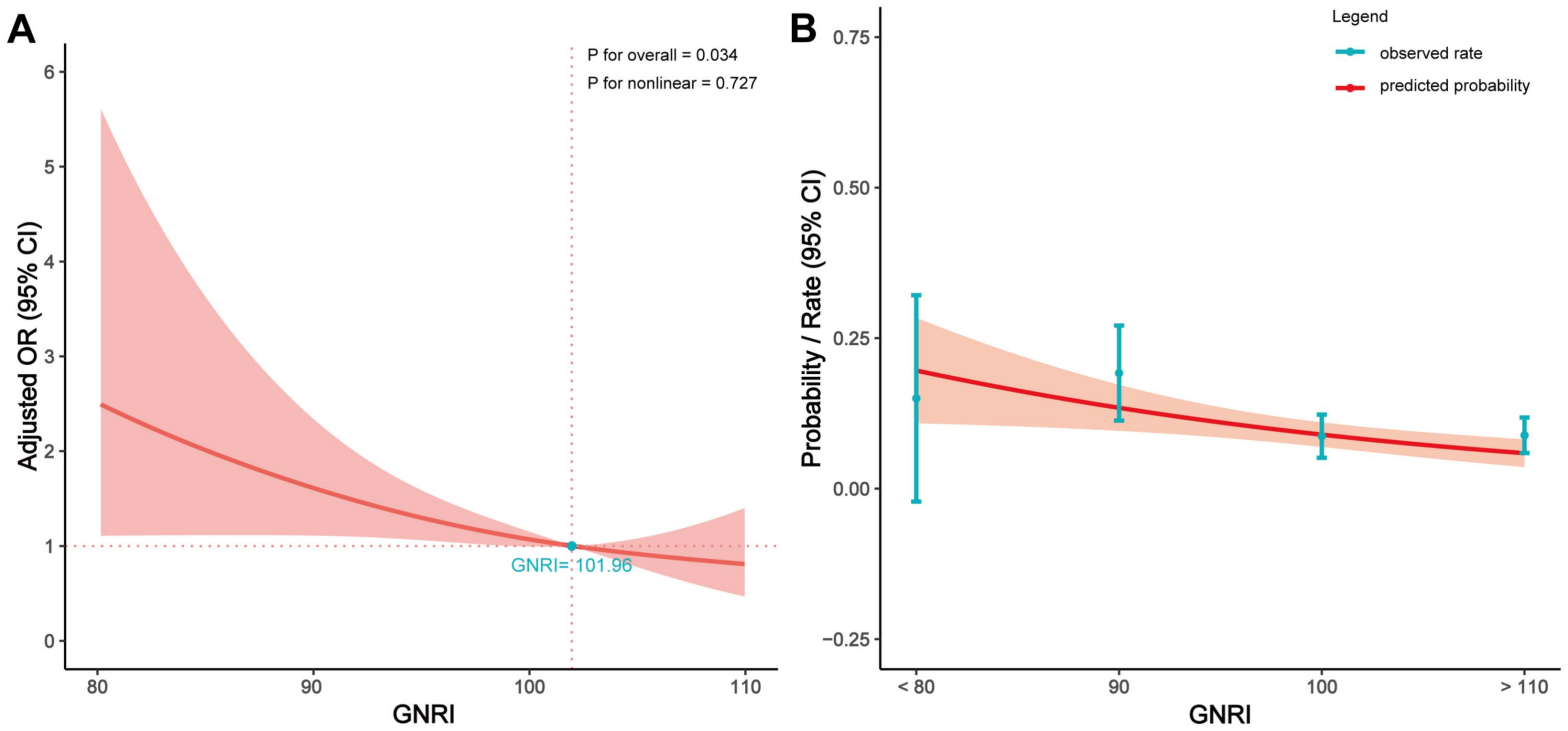
Relationship between the GNRI value and POD in patients of RTHA and RTKA. **(A)** Adjusted OR for POD according to values of GNRI; **(B)** Predicted probabilities and the observed rate of POD. The multivariate model was adjusted for BMI, COPD, valvular heart disease, cerebrovascular, solid-tumor, depression, VTE, admission to surgery, age, sex, surgery time, length of stay, and surgical site. OR, odds ratio; CI, confidence interval; GNRI, geriatric nutritional risk index.

Figure 3A further illustrates the relationship between preoperative GNRI values and POD. After adjusting for several covariates (including BMI, chronic obstructive pulmonary disease (COPD), valvular heart disease, cerebrovascular, solid-tumor, depression, VTE, admission to surgery, age, sex, surgery time, length of stay, surgical site), the results showed that a higher preoperative GNRI was significantly associated with a lower risk of POD (p for overall = 0.034, p for nonlinear = 0.727). When preoperative GNRI exceeded 101.96, the protective effect of GNRI surpassed its potential negative impact. Figure 3B displays the relationship between preoperative GNRI values and the predicted probability of POD, indicating that higher preoperative GNRI values correspond to a lower incidence of POD.

### Sensitivity and diagnostic efficacy analysis

**Figure 4:**
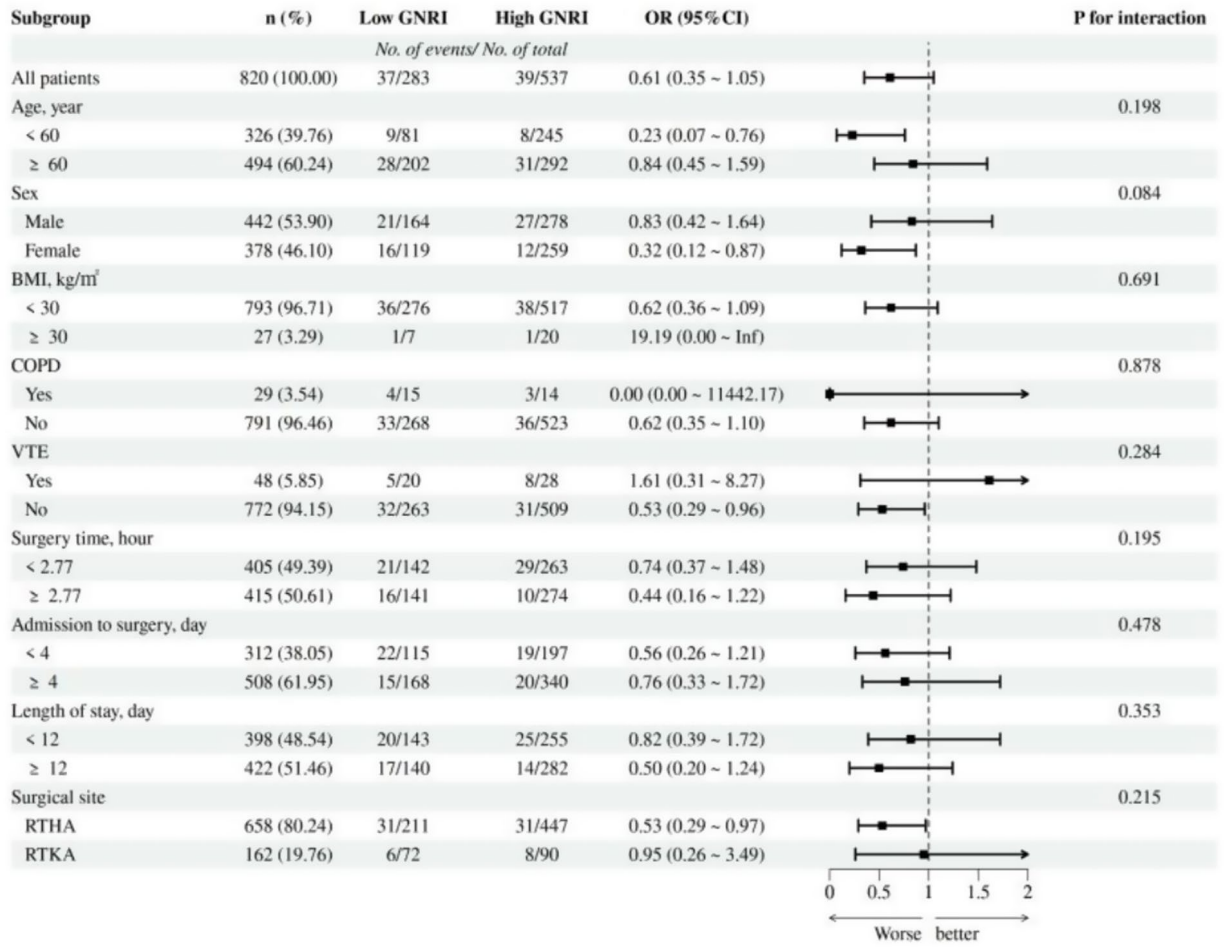
Subgroup analysis of the association between GNRI and POD. OR, odds ratio; CI, confidence interval; BMI, body mass index; COPD, chronic obstructive pulmonary disease; VTE, venous thromboembolism.

Moreover, we assessed the interactions among various factors affecting POD (Figure 4). The results indicated that the relationship between GNRI and POD was not significantly influenced by covariate subgroups (all p for interaction >0.05), suggesting that the relationship between preoperative GNRI and POD was quite stable. Lastly, the effectiveness of GNRI as a predictive tool for POD was evaluated using the ROC curve (Supplementary Figure 1), which revealed that the AUC for GNRI was 0.602, indicating a moderate predictive ability of GNRI in predicting POD.

## Discussion

This study is the first to apply GNRI to patients undergoing hip or knee revision arthroplasty, finding a significant linear dose–response relationship between preoperative GNRI values and the occurrence of POD among these patients. Notably, we identified a critical threshold value of GNRI at 101.96, above which GNRI exhibited a protective effect against POD. For every 1-unit increase in GNRI, the risk of POD decreased by 4%.

These findings are consistent with previous research. Chen et al.’s retrospective study reported a significant association between GNRI and POD, with an AUC for GNRI in the diagnosis of POD at 0.738 (95% CI: 0.660–0.817), sensitivity of 66.0%, and specificity of 70.4% (16). Although the AUC value in our study was lower (0.602), GNRI remains clinically relevant due to its simplicity, objectivity, and ease of use. It requires only routinely collected parameters—serum albumin, weight, and height—without the need for specialized equipment or subjective assessments. Thus, we believe GNRI can serve as a pragmatic, preliminary screening tool to identify elderly patients at elevated risk of POD, particularly in resource-limited settings or in early-stage perioperative evaluations. Research from the Medical Information Mart for Intensive Care (MIMIC) database also indicated a negative correlation between preoperative GNRI and POD, suggesting the inclusion of GNRI in POD predictive models to improve accuracy (10). Additionally, a large-scale retrospective study on surgical patients noted a nonlinear relationship between GNRI and POD, with a significant reduction in POD risk when GNRI exceeded 94 (30). A meta-analysis involving 4,242 patients by Xie et al. similarly confirmed that patients with malnutrition have a significantly higher risk of POD compared to nutritionally normal patients (OR = 2.04, 95% CI: 1.58–2.64, *p* < 0.001) (11). However, contrasting our findings, a prospective cohort study by Zhao et al. found no significant association between GNRI and POD in non-cardiac surgery patients (31). This discrepancy may stem from their smaller sample size (only 288 participants) and heterogeneity in study subjects (including various types of surgeries) (31).

Despite existing evidence indicating an association between GNRI and POD, the precise mechanisms remain unclear. Malnutrition can induce a catabolic state by reducing protein synthesis and accelerating metabolic degradation, leading to impaired immune function, which subsequently increases the risk of postoperative infections and inflammatory responses (13, 32). These inflammatory responses are characterized by elevated levels of inflammatory markers, such as interleukins (IL-6, IL-8, IL-10), tumor necrosis factor-alpha, S-100 calcium-binding protein beta, and C-reactive protein (33). Inflammation not only causes changes in peripheral blood but also affects the central nervous system, altering neurotransmission through the activation of microglia and astrocytes, disrupting the blood–brain barrier, and further impacting cognitive function (34, 35).

While this study focused on the GNRI, alternative nutritional indices such as the Prognostic Nutritional Index (PNI) have also been used to assess perioperative nutritional status{s10}. PNI is calculated based on serum albumin and total lymphocyte count, and has been associated with various postoperative outcomes, including complications and mortality{s11.s12.s13}. However, PNI requires differential blood counts, which may not be consistently available preoperatively in all institutions. In contrast, GNRI is derived solely from serum albumin and anthropometric data, making it simpler and more feasible for routine screening, especially in resource-limited settings. Nevertheless, the lack of direct comparison between GNRI and other nutritional indices, such as PNI or CONUT, represents a limitation of this study and warrants future research to determine the most effective predictive tool for POD.

This study suggests that GNRI not only reflects overall nutritional status but can also serve as an early screening tool to identify high-risk patients. Given the stability and effectiveness of GNRI in predicting POD, we recommend its incorporation into routine preoperative assessments to ensure the early identification of patients with poor nutritional status and to develop individualized intervention strategies, such as optimizing nutritional support and psychological interventions, to reduce the incidence of POD.

Interestingly, our study found that a longer waiting time from admission to surgery was significantly associated with a lower risk of postoperative delirium. One possible explanation is that extended preoperative time allows for better physiological stabilization and optimization of comorbid conditions, nutritional status, and medication management-factors that are particularly relevant in elderly patients undergoing revision arthroplasty. This period may also provide more time for multidisciplinary preoperative assessment and patient education, which have been linked to reduced POD incidence in previous literature. However, this finding should be interpreted with caution, as the association may also be influenced by unmeasured confounding, such as selection bias (e.g., healthier patients being scheduled later) or varying thresholds for surgical urgency. Future prospective studies are needed to confirm this observation and explore the causal relationship between surgical timing and POD risk.

Our subgroup analysis suggested a potential sex-related difference in the association between GNRI and POD. Specifically, female patients with higher GNRI exhibited a significantly lower risk of POD (OR = 0.32, 95% CI: 0.12–0.87), whereas the association in male patients was not statistically significant (OR = 0.83, 95% CI: 0.42–1.64). Although the P for interaction was 0.08, just above the conventional threshold for this finding may indicate that the protective effect of higher nutritional status is more pronounced in female patients. Possible explanations could include sex-related differences in nutritional reserve, inflammatory responses, and cognitive vulnerability. Further studies are warranted to investigate the biological or behavioral mechanisms underlying this differential effect and to explore whether sex-specific nutritional interventions could enhance perioperative care.

To our knowledge, this is the first study to evaluate the association between preoperative GNRI and POD specifically in patients undergoing revision hip or knee arthroplasty. Most previous studies have focused on primary joint replacements or other surgical populations, while evidence in the revision setting remains limited. Revision arthroplasty patients often present with greater physiological frailty, complex comorbidities, and a higher risk of postoperative complications. Therefore, our findings fill an important gap by highlighting a simple and objective nutritional assessment tool-GNRI-as a potential predictor of POD in this high-risk population. Given the increasing volume and complexity of revision procedures globally, integrating GNRI into preoperative evaluation protocols may help guide early risk stratification and improve perioperative management.

This study has several strengths, including a large sample size (820 patients) and rigorous data analysis. We utilized multivariate regression models to adjust for potential confounding factors, ensuring an accurate assessment of the independent association between GNRI and POD. By analyzing GNRI as both a continuous and categorical variable, along with quartile grouping, we precisely evaluated the relationship between GNRI and POD, further enhancing the reliability of our conclusions.

Nonetheless, this study does have limitations. First, since the study only included patients undergoing hip or knee revision arthroplasty, the generalizability of the results may be limited, and future research should extend to other surgical types. Second, as a single-center study, the external validity is constrained; future multi-center studies will help validate these results. Third, because this study was conducted at a single tertiary center in China with a relatively homogenous ethnic population, caution is warranted when extrapolating these results to Western or multiethnic populations. Differences in genetic background, cultural factors, dietary habits, and healthcare systems may influence both nutritional status and delirium risk. Additionally, our cohort included only patients undergoing revision arthroplasty, and the findings may not generalize to other surgical procedures with distinct perioperative risk profiles. Future multicenter studies involving more diverse patient populations and surgical types are needed to validate and extend the applicability of these findings. Fourth, as this study is observational in nature, the level of evidence is inherently limited. In particular, because the study was retrospective, we were unable to reliably determine whether any patients received preoperative nutritional interventions that could have affected their GNRI values or subsequent POD outcomes. No standardized nutritional interventions were implemented during the preoperative period, and any nutritional support provided was part of routine clinical care and not systematically recorded in the medical charts. This lack of documentation introduces the potential for unmeasured confounding, as we could not assess the extent or impact of preoperative nutritional support. This limitation underscores the need for future prospective studies that include controlled nutritional strategies and documentation of their impact on delirium risk. Fifth, data on baseline cognitive function and postoperative pain, recognized risk factors for POD-were not available due to the lack of standardized assessments and documentation in the electronic medical records. To address this limitation, we adjusted for relevant surrogate variables such as age, sex, depression, cerebrovascular disease, and surgical site, which have been shown to correlate with cognitive vulnerability and pain sensitivity (36–38). Moreover, the relationship between GNRI and POD remained consistent across subgroup and interaction analyses, suggesting a stable association despite the absence of direct measures. Future studies should incorporate formal cognitive screening and standardized pain assessments to improve confounding control and model accuracy.

Together, these limitations highlight the importance of future prospective, multicenter studies with comprehensive variable collection to further clarify the role of preoperative nutritional status in predicting postoperative delirium.

## Conclusion

This study indicates a significant association between the GNRI and POD. Specifically, patients with lower GNRI values are at a higher risk of developing delirium postoperatively, suggesting that GNRI has potential as a predictive tool for POD. Further research and clinical trials will contribute to developing more effective interventions for the postoperative management of elderly patients, thereby advancing the implementation of preoperative assessments and personalized treatments.

## Data Availability

The raw data supporting the conclusions of this article will be made available by the authors, without undue reservation.
