# Supplementary material for: Preoperative geriatric nutritional risk index as a predictor of postoperative delirium in revision arthroplasty: a 10-year retrospective cohort study
Source: Front Med (Lausanne). 2025 Jul 16;12:1626383. doi: 10.3389/fmed.2025.1626383 (PMC12307334; doi:10.3389/fmed.2025.1626383)
Supplement: Supplementary file 1 [file Data_Sheet_1.docx]

**eTable 1: List of variables included in the logistic regression full model.**

| Demographic |
| --- |
| Age |
| Gender |
| BMI |
| Education |
| Smoking |
| Alcohol abuse |
| Comorbidities |
| AHRQ ECI* |
| 1. Congestive heart failure |
| 1. Cardiac arrhythmias |
| 1. Valvular heart disease |
| 1. Pulmonary circulation disorders |
| 1. Peripheral vascular disorders |
| 1. Hypertension, uncomplicated |
| 1. Hypertension, complicated |
| 1. Paralysis |
| 1. Other neurological disorders |
| 1. Chronic pulmonary disease |
| 1. Diabetes, uncomplicated |
| 1. Diabetes, complicated |
| 1. Hypothyroidism |
| 1. Renal failure |
| 1. Liver disease |
| 1. Peptic ulcer disease, excluding bleeding |
| 1. AIDS / HIV |
| 1. Lymphoma |
| 1. Metastatic cancer |
| 1. Solid tumor without metastasis |
| 1. Rheumatoid arthritis/collagen vascular diseases |
| 1. Coagulopathy |
| 1. Obesity |
| 1. Weight loss |
| 1. Fluid and electrolyte disorders |
| 1. Blood loss anemia |
| 1. Deficiency anemia |
| 1. Alcohol abuse |
| 1. Drug abuse |
| 1. Psychoses |
| 1. Depression |
| Operation |
| Surgery time |
| Admission to surgery |
| Length of stay |
| Surgical site |
| ASA Classification |
| Nutritional Assessment Indicators |
| Albumin |
| GNRI |

Abbreviations: BMI, body mass index; AHRQ, Agency for Healthcare Research and Quality; ECI, Elixhauser comorbidity index; AIDS, acquired immunodeficiency syndrome; HIV, human immunodeficiency virus; ASA, the American Society of Anesthesiologists physical status classification system; GNRI, geriatric nutritional risk index. *The AHRQ ECI is calculated based on the incidence of these comorbidities in patients from 1 to 31.

**eTable 2: Baseline characteristics of the 820 patients by GNRI.**

| Characteristics | Total | Q1 | Q2 | Q3 | Q4 | *p*-value |
| --- | --- | --- | --- | --- | --- | --- |
|  |  | 62.69 - 95.15 | 95.16 - 102.00 | 102.01 - 107.22 | 107.23 - 122.70 |  |
|  | n = 820 | n = 205 | n = 203 | n = 205 | n = 207 |  |
| Demographic |  |  |  |  |  |  |
| Age, year (Mean ± SD) | 61.45 ± 14.32 | 64.44 ± 14.76 | 63.31 ± 13.82 | 59.78 ± 14.65 | 58.30 ± 13.21 | < 0.001* |
| Female gender (n, %) | 442 (53.90) | 120 (58.54) | 103 (50.74) | 112 (54.63) | 107 (51.69) | 0.385 |
| BMI, kg/m^2^ (Mean ± SD) | 23.64 ± 3.62 | 22.15 ± 3.93 | 23.46 ± 3.31 | 24.16 ± 3.50 | 24.79 ± 3.15 | < 0.001* |
| Education (n, %) |  |  |  |  |  |  |
| Illiteracy | 50 (6.10) | 19 (9.27) | 6 (2.96) | 12 (5.85) | 13 (6.28) | 0.001* |
| Below the junior high school | 200 (24.39) | 60 (29.27) | 55 (27.09) | 31 (15.12) | 54 (26.09) |  |
| Secondary education | 518 (63.17) | 114 (55.61) | 135 (66.50) | 148 (72.20) | 121 (58.45) |  |
| College degree and higher | 52 (6.34) | 12 (5.85) | 7 (3.45) | 14 (6.83) | 19 (9.18) |  |
| Smoking (n, %) | 189 (23.05) | 33 (16.10) | 57 (28.08) | 55 (26.83) | 44 (21.26) | 0.015* |
| Alcohol abuse (n, %) | 170 (20.73) | 36 (17.56) | 51 (25.12) | 44 (21.46) | 39 (18.84) | 0.245 |
| Comorbidities |  |  |  |  |  |  |
| AHRQ ECI (Mean ± SD) | 2.03 ± 6.44 | 4.04 ± 7.34 | 2.03 ± 6.85 | 1.30 ± 5.57 | 0.77 ± 5.36 | < 0.001* |
| Operation |  |  |  |  |  |  |
| Surgery time, hours (Mean ± SD) | 2.68 ± 0.67 | 2.71 ± 0.73 | 2.81 ± 0.64 | 2.69 ± 0.54 | 2.50 ± 0.73 | < 0.001* |
| Admission to surgery, day (Mean ± SD) | 5.35 ± 4.54 | 5.38 ± 5.15 | 5.66 ± 4.11 | 5.78 ± 5.14 | 4.57 ± 3.51 | 0.032* |
| Length of stay, day (Mean ± SD) | 13.99 ± 10.35 | 14.48 ± 13.11 | 15.38 ± 10.51 | 13.89 ± 8.90 | 12.26 ± 7.99 | 0.019* |
| Surgical site (n, %) |  |  |  |  |  |  |
| RTHA | 658 (80.24) | 154 (75.12) | 161 (79.31) | 166 (80.98) | 177 (85.51) | 0.066 |
| RTKA | 162 (19.76) | 51 (24.88) | 42 (20.69) | 39 (19.02) | 30 (14.49) |  |
| ASA Classification (n, %) |  |  |  |  |  |  |
| Ⅰ - Ⅱ | 380 (46.34) | 82 (40.00) | 75 (36.95) | 102 (49.76) | 121 (58.45) | < 0.001* |
| Ⅲ - IV | 440 (53.66) | 123 (60.00) | 128 (63.05) | 103 (50.24) | 86 (41.55) |  |
| Nutritional Assessment Indicators |  |  |  |  |  |  |
| Albumin, g/L (Mean ± SD) | 40.59 ± 5.59 | 33.48 ± 4.23 | 39.31 ± 2.18 | 42.73 ± 1.49 | 46.77 ± 2.14 | < 0.001* |
| GNRI, (Mean ± SD) | 100.72 ± 8.97 | 88.60 ± 6.03 | 98.75 ± 1.99 | 104.50 ± 1.47 | 110.94 ± 2.92 | < 0.001* |
| Outcome |  |  |  |  |  |  |
| Non-POD | 744 (90.73) | 173 (84.39) | 191 (94.09) | 187 (91.22) | 193 (93.24) | 0.003* |
| POD | 76 (9.27) | 32 (15.61) | 12 (5.91) | 18 (8.78) | 14 (6.76) |  |

Abbreviations: GNRI, geriatric nutritional risk index; SD, standard deviation; BMI, body mass index; AHRQ, Agency for Healthcare Research and Quality; ECI, Elixhauser comorbidity index; RTHA, revisional total hip arthroplasty; RTKA, revisional total knee arthroplasty; ASA, the American Society of Anesthesiologists physical status classification system; POD, postoperative delirium*.* * *p* < 0.05.


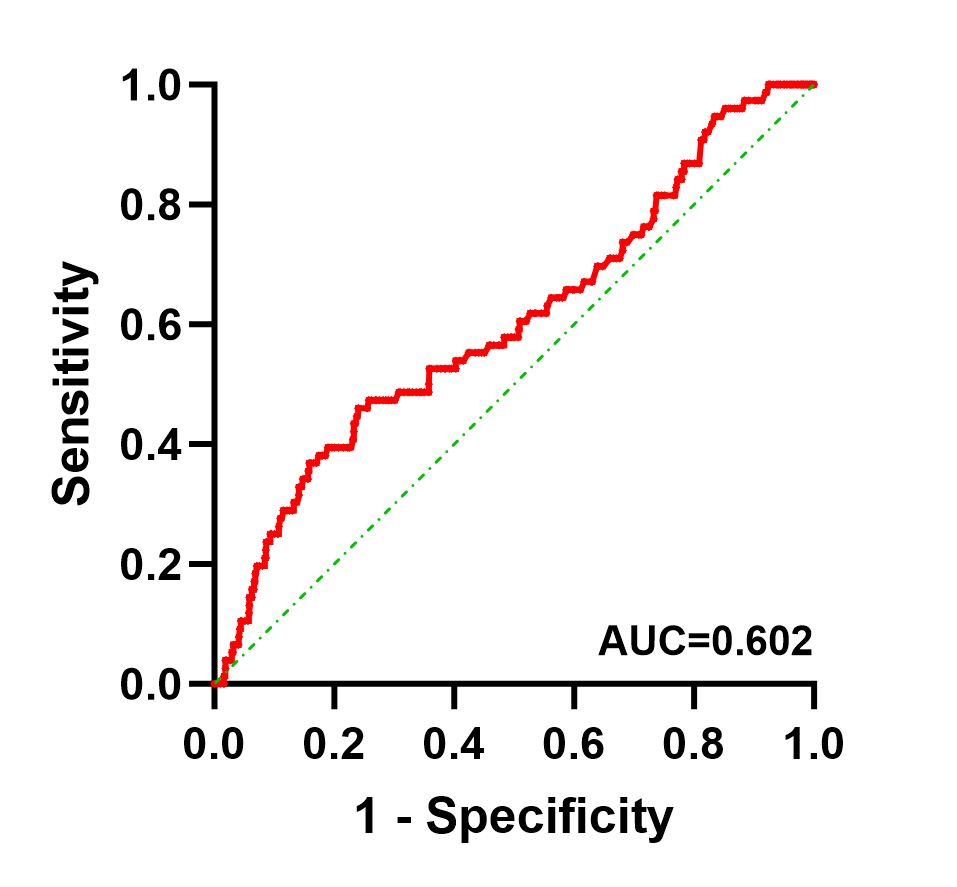


**eFigure 1:** **ROC curve analysis to evaluate the predictive value of GNRI for POD in patients of RTHA and RTKA.** ROC, receiver operating characteristic; AUC, the area under the curve.
